# Computer aided protein engineering to enhance the thermo-stability of CXCR1- T4 lysozyme complex

**DOI:** 10.1038/s41598-019-41838-2

**Published:** 2019-03-29

**Authors:** Yang Wang, Jae-Hyun Park, Cecylia Severin Lupala, Ji-Hye Yun, Zeyu Jin, Lanqing Huang, Xuanxuan Li, Leihan Tang, Weontae Lee, Haiguang Liu

**Affiliations:** 1 Complex Systems Division, Beijing Computational Science Research Center, Beijing, 100193 China; 2 Department of Biochemistry, College of Life Science & Biotechnology, Yonsei University, Seoul, 03722 South Korea; 3 Department of Engineering Physics, Tsinghua University, Beijing, 100084 China; 4 Department of Physics and Institute of Computational and Theoretical Studies, Hong Kong Baptist University, Hong Kong, China

## Abstract

CXCR1, a member in G-protein coupled receptor (GPCR) family, binds to chemokine interleukin-8 (IL-8) specifically and transduces signals to mediate immune and inflammatory responses. Despite the importance of CXCR1, high-resolution structure determination is hindered by the challenges in crystallization. It has been shown that properly designed mutants with enhanced thermostability, together with fusion partner proteins, can be useful to form crystals for GPCR proteins. In this study, *in silico* protein design was carried out by using homology modeling and molecular dynamics simulations. To validate the computational modeling results, the thermostability of several mutants and the wild type were measured experimentally. Both computational results and experimental data suggest that the mutant L126W has a significant improvement in the thermostability. This study demonstrated that *in silico* design can guide protein engineering and potentially facilitate protein crystallography research.

## Introduction

Chemokine molecules and their interactions with receptors are crucial to cellular immunity, cancer and inflammation regulations^1,2^. Chemokine receptors belong to the superfamily of G-protein coupled receptors (GPCRs), which are major targets for drug design. There are about 45 chemokines and 22 chemokine receptors in human bodies, forming a pharmacologically active complex system^3^. Currently there are several compounds targeting chemokine receptors in clinical trials for the treatment of cancer, chronic obstructive pulmonary disease (COPD), asthma, rheumatoid arthritis, and HIV infections^4^.

Chemokine molecules share a common topology, composed of a flexible N terminal with a conserved double-cysteine motif, followed by a single turn of 3_10_ helix, a three-strand-β-sheet, and a helix domain at C-terminal^5^. The nomenclature of chemokines and their receptors is based on the sequence of cysteine residues in chemokines^6^. The pattern of cysteine residues in the N terminal serves as a basis for chemokine classification to CC, CXC and CX_3_C chemokines with the cysteine residues separated by zero, one, and three residues respectively.

**Figure 1:**
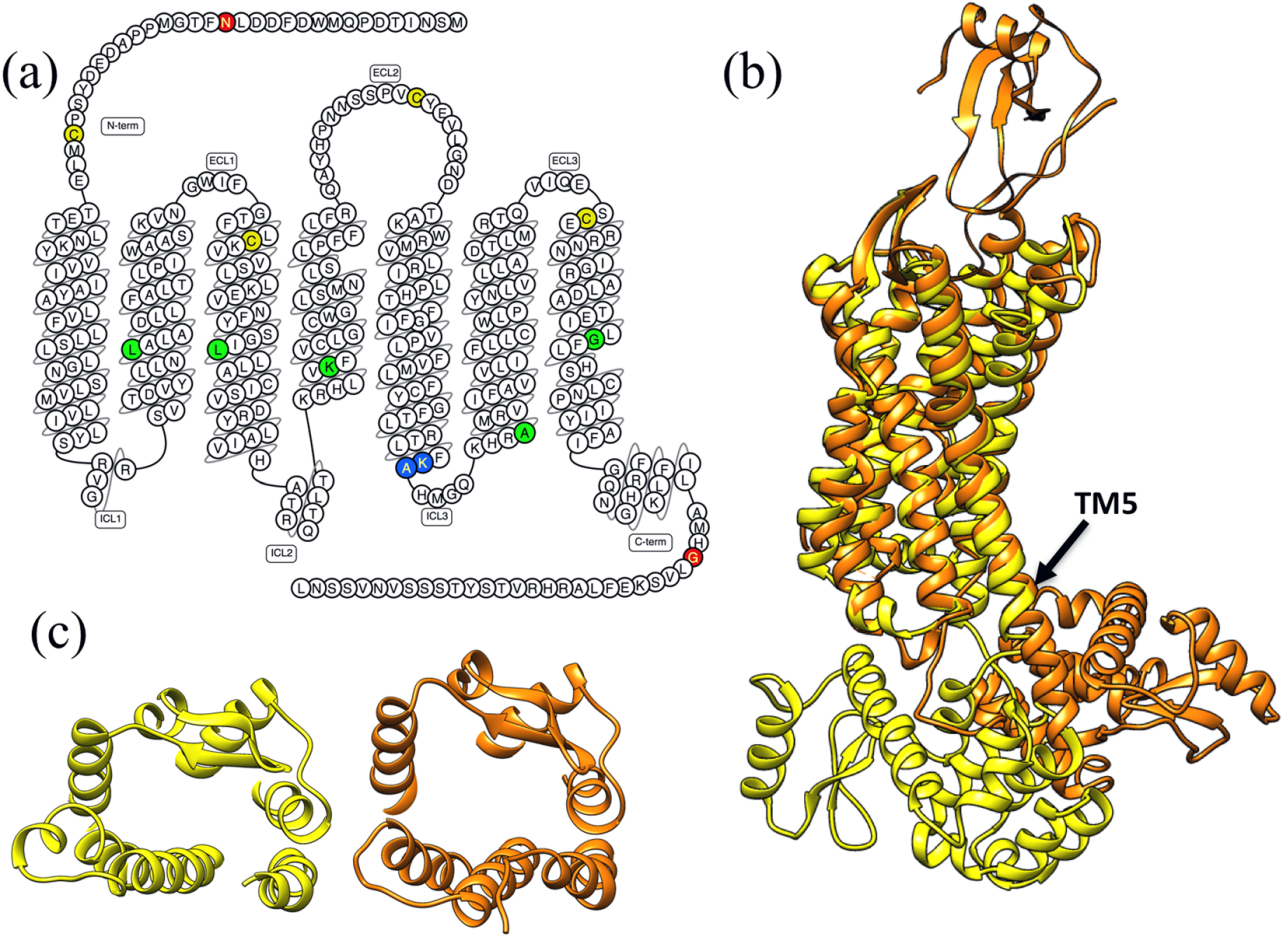
CXCR1 protein and structural templates. (**a**) Topology and primary structure of CXCR1, with the residues subject to mutation colored in green. The snake plot was drawn with GPCRdb^17^. (**b**) The structure comparison of two homology template structures: CCR2-T4L (PDB ID: 5T1A, colored in yellow) and the CXCR4-T4L (PDB ID: 4RWS, colored in brown). The black arrow points to the TM5, where the helix conformation extends to the T4L domain in the case of CXCR4-T4L. (**c**) The structure comparison of CCR2-T4L and CXCR4-T4L at the extracellular domain reveals the CXCR4-T4L binding pocket for ligand (vMIP-II, not shown) is in the open conformation.

CXCR1 is one of the two high-affinity receptors for the CXC chemokine interleukin-8 (IL-8). Although the IL8 was the first chemokine with experimentally determined structure^7^, the structural knowledge of its receptor CXCR1 remained limited, until the first model of CXCR1 (PDB ID: 2LNL) was solved using solid state NMR method^8^. As shown in Fig. 1a, the topology of CXCR1 is composed of seven trans-membrane helices (TM1-TM7), three extracellular loops (ECL1-ECL3) and three intracellular loops (ICL1-ICL3). There are two disulfide bonds holding the heptahelical bundle of CXCR1 molecule. One disulfide bond connects TM3 to ECL2 while the other disulfide bond links N terminal domain to ECL3. Although the NMR structure ensembles provide valuable insights about the CXCR1 structure and functional basis, the binding pocket is mainly buried and difficult for computational ligand docking to the binding site. Therefore, a high resolution of CXCR1 structure determined using crystallography method is highly desired.

GPCRs are known to be difficult to crystallize, mainly because of their transmembrane nature. Thermo-stabilizing mutations, truncations of flexible terminal residues, as well as insertion of the protein T4 lysozyme (T4L), have facilitated the crystallization of GPCR proteins and their structure determinations using crystallography methods. Furthermore, addition of antibodies or small molecules can improve structure stability and the molecular contacts in crystalline state^9^. The structures of CXCR4 (PDB ID: 4RWS) in CXC chemokine receptor subfamily and CCR2 (PDB ID: 5T1A) in the CC chemokine receptor subfamily have been resolved using crystallography methods^10,11^. Both CXCR4 and CCR2 were crystallized with T4L as the fusion partner protein, and CXCR4-T4L was also co-crystallized with the antagonist chemokine vMIP-II. The receptors in the Chemokine receptor family have high sequence similarity, especially in trans-membrane (TM) regions. The sequence similarity between CXCR1 and CXCR4 is about 38% for full sequence and about 42% for transmembrane regions; while CXCR1 and CCR2 share a sequence similarity of about 33% for full sequence (about 38% for transmembrane regions). Therefore it is possible to predict the 3D structure of CXCR1 using homology modeling methods^12,13^. Interestingly, the crystal structures of CXCR4 and CCR2 are very similar, with an RMSD (root mean square deviation) of less than 3 Å for TM regions, while the CXCR1 structure resolved using solid state NMR method has an RMSD of about 5 Å compared to either CXCR4 or CCR2 structures. These three structures share the same topology with the main differences in the loop regions and helix packing in the TM cores. Because the NMR structure of CXCR1 does not include the T4L domain, we took the CXCR4 and CCR2 crystal structures as templates to build homology models in this study. These two structure templates provide complementary information: CXCR4-T4L complex does not have the atomic coordinates for C terminal residues, while the CCR2-T4L complex lacks the structural information at the N terminal. In this study, based on these two template structures, the CXCR1-T4L complex structure was predicted using I-TASSER and MODELLER^14–16^.

Starting with the homology models, the mutants that can improve thermostability were investigated. Based on the homology models, the tools in GPCR Data Base (GPCRdb) were used to guide the design of mutant constructs for CXCR1-T4L complex^17,18^. The truncations of CXCR1 at both N/C termini, the insertion sites for fusion protein T4L, and amino acid mutations were carefully selected with the aim to improve the thermostability. The 3D structure for each mutant and wild type construct was subjected to extensive all-atom molecular dynamics simulation to characterize the stability. The thermostability was also measured experimentally, and the results were compared with the data from computational modeling and simulation. Compared to the wild type, two mutants lead to enhanced thermostability, especially the L126W mutant improved the melting temperature by 8.37 °C.

## Methods

### Construct design for CXCR1-T4L complex

The overall topology of CXCR1 is shown in Fig. 1a in the form of primary structure, with key residues highlighted using different colors. T4L protein is the most frequently used fusion partner to improve the stability and molecular contacts. It is interesting to find that nearly 41% of solved GPCR structures were determined by inserting T4L into ICL3 (intracellular loop 3)^9^. Inspired by this fact, the T4L with a three-residue SGS linker added to its C-terminal was inserted to ICL3 between K231^5.67^ and A232^5.68^ (see Fig. 1a for details). The N terminal of CXCR1 was truncated to N16, for two reasons: (1) the N-terminal is likely to be disordered as there is no structural information in either CXCR4 or CCR2 templates; and (2) the binding between CXCR1 N-terminal and the chemokine IL8 is mainly achieved via the electrostatic interactions between the K11, K15, R47 of the chemokine IL8 and the D24, E25, D26 of the CXCR1 N-terminal residues^7^. The C-terminal amino acids beyond G324 of CXCR1 were also removed under similar considerations. Since this study aims to investigate the stability of the CXCR1 core region in the presence of T4L fusion partner, the truncations at termini are expected to cause insignificant differences.

### Homology modeling for CXCR1-T4L

Three structures, the NMR structure of CXCR1 (PDB ID: 2LNL)^8^, the crystal structure of CXCR4-T4L-vMIP-II complex (PDB ID: 4RWS)^10^, and the crystal structure of CCR2-T4L (PDB ID: 5T1A)^11^, provide templates to build the CXCR1-T4L complex structure. The TM region of CXCR4 and CCR2 templates have similar backbone structures (within 3 Å RMSD), and the NMR model for CXCR1 shows larger deviation from these two structures. Between CXCR4-T4L and CCR2-T4L structures, the main differences are the relative position of the fusion partner T4L (Fig. 1b). The extracellular side of CXCR4-T4L complex exhibits an open conformation compared to the structure of CCR2-T4L (Fig. 1c). This conformation allows the binding of viral chemokine antagonist vMIP-II (the open conformation might be induced by the binding of vMIP-II). It is also worthwhile to point out that the helical conformation of TM5 in CXCR4 structure extends to T4L domain, forming a longer helix than the other six TM domains. For the structure of CCR2-T4L complex, the TM5 helix is linked to T4L via a flexible loop.

**Figure 2:**
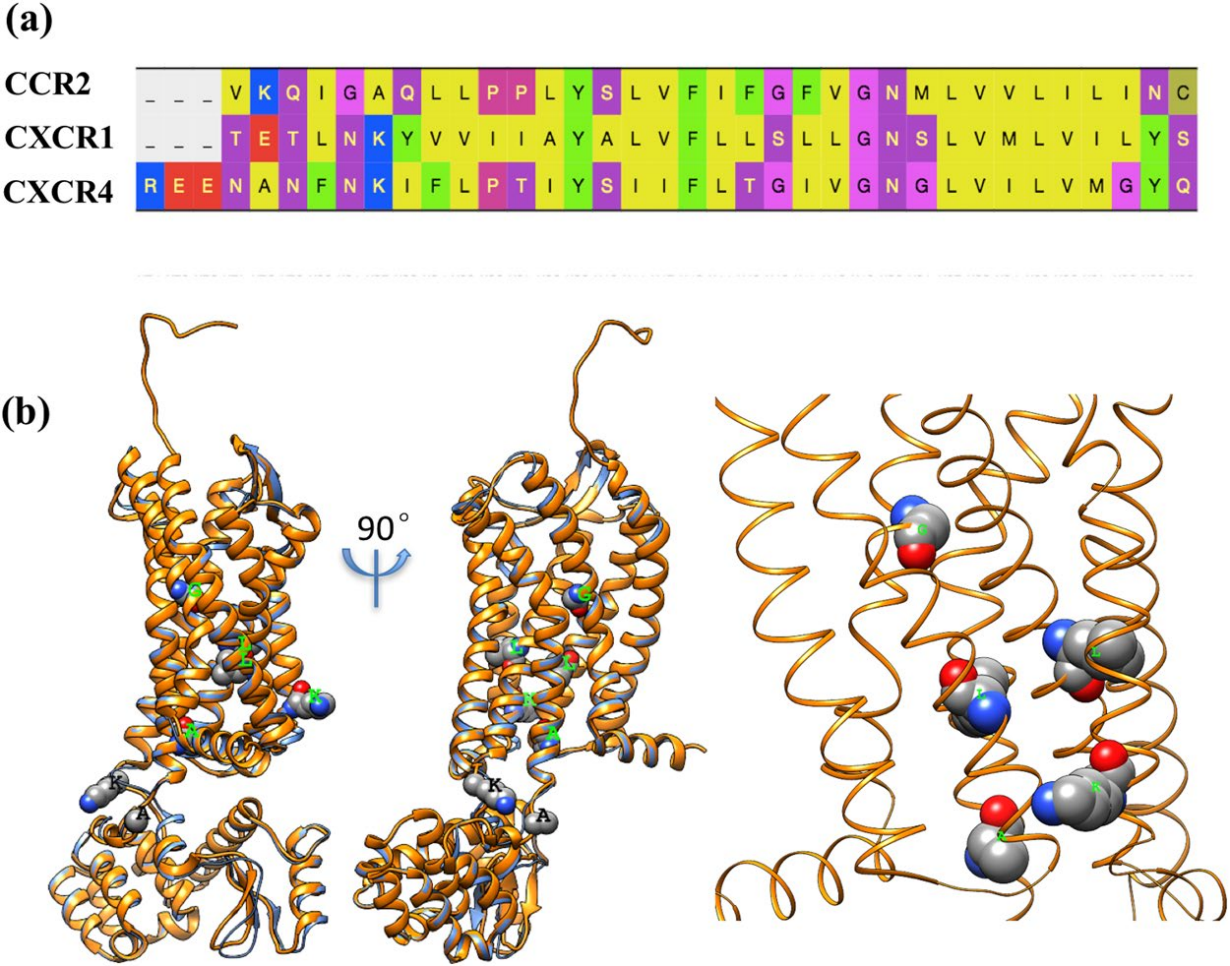
Sequence alignment and homology modeling results. (**a**) Sequence alignment of CCR2, CXCR1 and CXCR4 in TM1. (**b**) The CXCR1-T4L structure predicted using CCR2-T4L homology modeling, with the N-terminal structure grafted from CXCR4-T4L template. The T4L was inserted between K231^5.67^ and A232^5.68^. The blue ribbon shows the template CCR2-T4L structure. The right panel shows the residues that are subjected to mutation.

Because of these differences in conformation and ligand binding states, we used the CCR2-T4L structure as the primary template to build the homology model, HM-CCR2 (Fig. 2b). In the crystal structure of CCR2-T4L complex, the conformations for N terminal residues were not resolved, so the N-terminal (N16-V41) was built based on the structure of CXCR4-T4L complex. This is justified by the fact that three proteins have high sequence similarity in N-terminal (Fig. 2a). The Modeler interface in Pymol (PyMod) was used for the structure grafting^16,19^.

### The design of CXCR1 mutant based on GPCRdb and the homology models

Experiments have shown that certain point mutations can enhance the molecular stability without significantly change the structure^20^. Therefore, the goal is to find these point mutants that enhance the thermostability, without directly perturbing overall structure or the ligand binding interface in the CXCR1-T4L complex. There are 37 recommended mutant constructs based on accumulated knowledge in the GPCRdb for the improved thermostability, with five principles under considerations, including homology, common mutations, conservation of mutation sites, helical propensity, and state switches (see the documentation of the GPCRdb for details). It is difficult to narrow down the recommended mutants without additional information. In this work, with the aid of homology models described in section 2.2, the following mutants were selected from the recommendation list: L81^2.46^A, L126^3.41^W, K154^4.43^A, A240^6.33^D, G294^7.42^A (See Table 1 for a summary about the potential impacts to the structure and stability of each mutation). In the following, we discuss the detailed interactions near the mutation site based on the 3D structure from homology modeling and the NMR structure of CXCR1, referred as HM-CCR2 and NMR-CXCR1 respectively, for clarity.

1. The side chain of L81^2.46^ is close to the side chain of L127^3.42^ in the HM-CCR2, forming a hydrophobic patch region, although the L81^2.46^ is close to the M61^1.54^ in the NMR-CXCR1 structure (Fig. 3a). Based on the homology model, the L81^2.46^A mutant may influence the hydrophobic interactions and affect the helical packing, as alanine usually enhances helical conformation.

**Figure 3:**
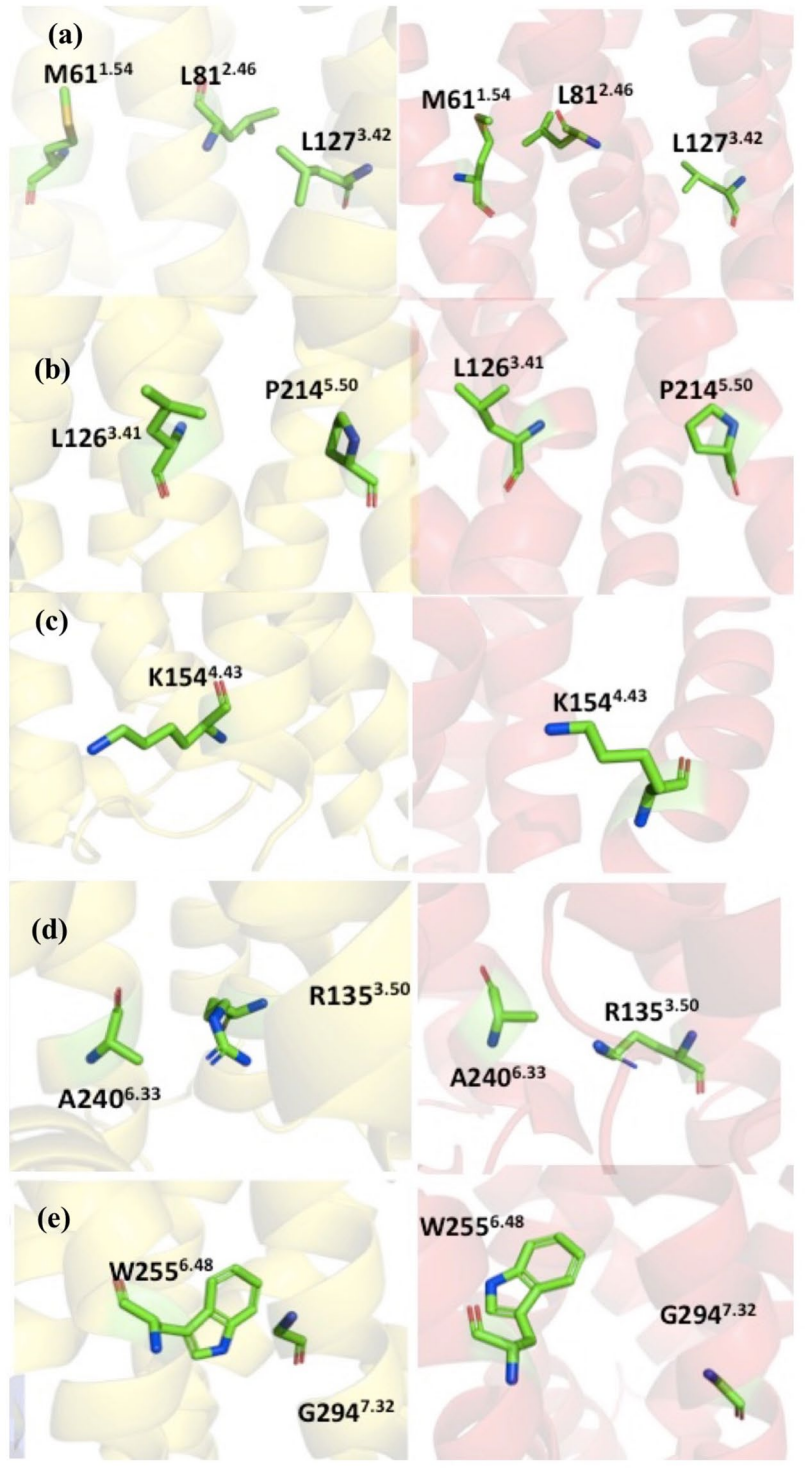
The mutational sites and interacting residues. The structures are colored as the following: HM-CCR2 (yellow), and the NMR-CXCR1 (red). The local structure near the mutation sites and the neighboring residues: (**a**) M61^1.54^, L81^2.46^ and L127^3.42^; (**b**) L126^3.41^ and P214^5.50^; (**c**) K154^4.43^; (**d**) R135^3.50^ and A240^6.33^; (**e**) W255^6.48^ and G294^7.32^.
2. As shown in Fig. 3b, L126^3.41^ and P214^5.50^ are next to each other in both HM-CCR2 and NMR-CXCR1 structures. The mutation of leucine to tryptophan can potentially enhance this interaction, and may also establish stacking of the aromatic rings.
3. In Fig. 3c, the side chain of K154^4.43^ points towards the membrane and does have other specific interactions with other nearby residues. The mutation of K154^4.43^A was aimed at promoted hydrophobicity in TM region.
4. Figure 3d shows the side chain of A240^6.33^ and neighboring residues. It is clear that A240^6.33^ is in the vicinity of R135^3.50^ in both structures. We designed the mutation of A240^6.33^D, to study whether it will enhance the structure stability by forming salt bridge interactions with R135^3.50^.
5. The side chains of G294^7.32^ and W255^6.48^ exhibit close contacts in HM-CCR2 structure (Fig. 3e). The mutation of G294^7.42^A can potentially increase the helicity of the TM7 and the helical packing with TM6 by interacting with W255^6.48^.

**Table 1:** Point mutation design.

Mutation	Segment	Interacting residues	Rationale for mutation
L81^2.46^A	TM2	M61^1.54^, L127^3.42^	Helical conformation and packing
L126^3.41^W	TM3	P214^5.50^	Distance between L126^3.41^ and P214^5.50^
K154^4.43^A	TM4	None	Increase hydrophobicity in TM region
A240^6.33^D	TM6	R135^3.50^	Interact with DRY motif in TM3
G294^7.32^A	TM7	W255^6.48^	Interact with CWxP motif in TM6

### Molecular dynamics simulation

To evaluate the impacts of point-mutation on the stability of the CXCR1-T4L complex, the wild type and L81^2.46^A, L126^3.41^W, K154^4.43^A, A240^6.33^D, G294^7.42^A mutants of CXCR1-T4L complex were constructed using all-atom models and their dynamics were simulated in explicit lipid-solvent environments. The PPM server was used to reorient the CXCR1-T4L structures to ensure that the residues in TM region of CXCR1 were well located in the DMPC (1,2-dimyristoyl-*sn*-glycero-3-phosphocholine) lipid bilayer^21^. Before long time scale production simulations, the systems were equilibrated in a DMPC lipid bilayer and then solvated in a water box with X Y Z dimension of 87 Å, 87 Å and 154 Å respectively. CHARMM-GUI was used to generate topology and parameter files with CHARMM36 force fields^22–24^. In addition to the CXCR1-T4L complex molecule, 200 DMPC molecules, about 24,000 water molecules, and an appropriate amount of excess sodium chloride ions were added to maintain an ion concentration of 150 mM.

The NPT ensemble (constant pressure and constant temperature) MD simulations were generated using GROMACS 5.1.2^25^. The REDUCE program in AMBER was used to add hydrogens to the original PDB files and determine the protonation state of histidine residues^26,27^. Initial energy minimizations were achieved using the steepest descent algorithm, followed by a two-stage equilibration, 20 ns NVT (constant particle number, volume and temperature) dynamics simulation with harmonic restraint forces applied to the complex molecules (4000KJ mol^−1^ nm ^−2^ on backbone and 2000 KJ mol^−1^ nm^−2^ on side chain), followed by a 40 ns NPT dynamics simulation with gradually decreased restraint forces (from 2000 KJ mol^−1^ nm^−2^ to 100 KJ mol^−1^ nm^−2^ on backbone and from 1000 KJ mol^−1^ nm^−2^ to 50 KJ mol^−1^ nm^−2^ on side chains). During equilibration, harmonic restraints were applied to heavy atoms of the protein complex, and planar restraints were used to keep the positions of lipid head groups along membranes Z-axis. The simulation temperature of the system was set to 303 K. Once all the equilibration steps were completed, the restraints were removed and three independent 500 ns trajectories starting from different random velocities following Maxwell distributions were generated for each system (wild type or mutants) in NPT ensembles with a time step of 2 fs. The cubic periodic boundary condition was used during simulation and the long-range van der Waals interaction cut-off was set to 12 Å.

### Protein expression and purification of CXCR1 and its mutants

The gene encoding human CXCR1 conjugated with thermal stable mutant of T4 Lysozyme (T4L, C54T, C97A) was amplified and was inserted into the modified pFastbac vector using two restriction enzymes, AscI and FseI. Site-specific mutations were introduced using standard Site-Directed mutagenesis PCR. Subcloned CXCR1 gene, including hemagglutinin signal peptide and flag-tag in N-terminus together with prescission protease cleavage site and 10 x Histidine in C-terminus, were transformed into DH10Bac^TM^ *E.coli* for transposition into the bacmid. The recombinant bacmids of wild type CXCR1 and its thermal stable mutants were isolated by ethanol precipitation method and transfected into Sf9 cells by Cellfectin reagent (Invitrogen). Transfected Sf9 cells were incubated at 27 °C and phase 1 viruses were harvested after 96 h. Protein was overexpressed using high-titer virus (MOI of 3), generated in phase 3, during 72 h at 27 °C.

Infected cells were harvested by centrifugation and washed 2 times by phosphate buffered saline (PBS) and flash-frozen with 10% glycerol before −80 °C storage.

Membranes were purified by repeated lysis and homogenization using hypotonic buffer (10 mM HEPES pH 7.5, 10 mM MgCl2, 20 mM KCl) and high salt buffer (1.0 M NaCl, 10 mM HEPES pH 7.5, 10 mM MgCl2, 20 mM KCl) with EDTA-free Protease Inhibitor (PI, Roche). Purified membranes were thawed on ice and resuspended in buffer containing 50 mM HEPES pH 7.5, 500 mM NaCl, 200 μM purified IL-8 and PI. Membranes were incubated at 4 °C for 1 h, then solubilized in 50 mM HEPES pH 7.5, 500 mM NaCl, 200 μM purified IL-8, 1% (w/v) n-dodecyl-β-D-maltopyranoside (DDM, Anatrace), 0.2% (w/v) cholesteryl hemisuccinate (CHS, Anatrace) for 3 h at 4 °C with tumbling. The supernatant was isolated by centrifugation at 370,000 × *g* for 1 h, and incubated with 5 ml of TALON IMAC resin overnight at 4 °C. The resin was washed with 10 column volumes (cv) of wash buffer I (50 mM HEPES pH 7.5, 800 mM NaCl, 10% (v/v) glycerol, 0.1% (w/v) DDM, 0.02% (w/v) CHS, 5 mM imidazole and 200 μM purified IL-8) and then washed 10 cv of wash buffer II (50 mM HEPES pH 7.5, 500 mM NaCl, 10% (v/v) glycerol, 0.05% (w/v) DDM, 0.01% (w/v) CHS, 100 μM purified IL-8). The protein was eluted by 3–5 cv of elution buffer (50 mM HEPES pH 7.5, 500 mM NaCl, 10% (v/v) glycerol, 0.05% (w/v) DDM, 0.01% (w/v) CHS and 300 mM imidazole) without purified IL-8. PD MiniTrap G-25 column (GE Healthcare) was used to remove imidazole and exchange buffer to 25 mM sodium phosphate, 100 mM NaCl, 0.03% (w/v) DDM, 0.006% (w/v) CHS. The protein was then concentrated to 500 μl with 100 kDa cut-off Vivaspin concentrator.

### Thermostability assay using circular dichroism (CD) spectroscopy

Circular dichroism (CD) spectroscopy was performed by purified CXCR1 and its mutants. Far-UV CD spectra were monitored using JASCO J-815 spectrometer. The path length was 2 mm, and instrument parameters were set to a sensitivity of ~30 millidegrees, a response time of 1 s, and a scan speed of 100 nm/min. Spectra were recorded as an average of 3 scans.

To estimate the melting temperature (T_m_), the fraction of denatured protein (Δ*f*_D_) was calculated as Δ*f*_D_ = (θ − θ_min_)/(θ_max_ − θ_min_), where θ is the ellipticity at a given temperature and the θ_min_ and θ_max_ are lowest and highest values at all temperature. T_m_ were calculated by difference of ellipticity values at a wavelength of 222 nm using the Hill equation.

### Analysis

The overall RMSD was calculated for all non-hydrogen atoms with the tools in Gromacs package. The figures were prepared with Chimera^28^ and VMD^29^ programs.

The 7 × 7 RMSD matrix was obtained by aligning target structure to the reference with respect to each of the 7 TM helices and computing the RMSD for all TM and its counterpart in the target structure^30^. The 7 × 7 RMSD matrix decomposed and magnified the structural differences between the reference and target structures into 49 parameters. The seven diagonal elements served as an indicator of conformational changes within the TM helices themselves; The off-diagonal elements at each row reveal whether the corresponding TM moved from the reference structure relatively to the other TMs, i.e., the packing of the TM bundle. In the calculation of 7 × 7 RMSD matrix, each alignment was carried out for backbone atoms in each TM domain using VMD scripts^29^.

The melting temperature was calculated by optimizing parameters of a sigmoid function fitting to experimental data.

## Results

The stability of each system (wild type and mutants) was investigated using all-atom molecular dynamics simulations. For each system, three independent 500 ns simulation trajectories were generated, resulting a structure ensemble of 1,500 structures for detailed analysis. We compared the structural fluctuation quantified using RMSD with respect to the average structure in each ensemble. The interactions between the key amino acids at mutation sites and their neighboring residues were assessed using distances between the centers of side chains. The clustering analysis of the structural ensemble was carried out to quantify the distributions of structures in each ensemble. These quantitative measures were then used to describe the stability differences due to mutations. The thermostability data measured experimentally were compared with the results from simulations.

### The conformational fluctuations

For the wild type and five mutants, the conformational fluctuation is quantified by the RMSD of compared to the average model in each structural ensemble. We used four RMSD values to measure the structural difference between each structure and the average model of the corresponding ensemble, specifically: (1) RMSD^TM^, considering only the differences in transmembrane domain of CXCR1; (2) RMSD^T4L^, measuring the structure difference in the T4L domain; (3) RMSD^ALL^, measuring the overall structural differences of the CXCR1-T4L complex; and (4) RMSD^T4L’^, similar to RMSD^T4L^, but the transformation matrix for RMSD^TM^ is applied to T4L domain prior to the difference computation, therefore, RMSD^T4L’^ quantifies the relative motion between T4L and CXCR1.

**Figure 4:**
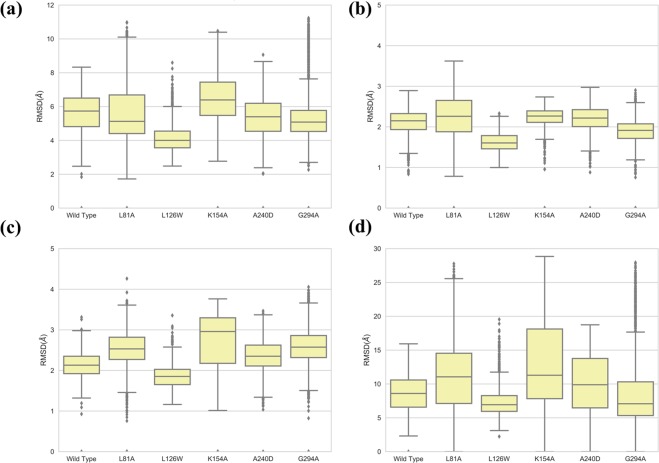
The RMSD^ALL^ (**a**), RMSD^TM^ (**b**), RMSD^T4L^ (**c**), and RMSD^T4L’^(**d**) from simulations using HM-CCR2 structures as initial conformations (see main text for details).

As described in the Method section, three independent trajectories were simulated for each construct with the HM-CCR2 homology structure as initial models. The aggregated simulation time is 1.5 microseconds for each system. From the simulation trajectories for each construct, 1,500 snapshot structures were selected with 1.0 ns separation to form the structure ensemble, from which the statistics were obtained. The RMSD analysis results were summarized in Fig. 4 for all six constructs. The box plots were used to present the RMSD distributions, where the data within 1.5 IQR (interquartile range) of the middle 50% were shown in the box with the median value, and the outliers were plotted with dots. The smaller median values indicate smaller deviation from average structure, and the smaller box size corresponds to narrower distributions. The RMSD results suggested that the CXCR1 transmembrane domain were stable for all constructs, with most median values of RMSD between 2 Å and 3 Å. Among the five mutant constructs, the L126W system showed the smallest fluctuations in the simulations starting with HM-CCR2 structure (Fig. 4). Pronounced motions of T4L relative to CXCR1 were observed in the mutants of L81A, K154A and A240D, resulting larger RMSD values. Based on the RMSD^TM^ statistics, the ranking of stability can be summarized as the following:

$${\rm{L126W}} > {\rm{G294A}} > {\rm{WT}} \sim {\rm{A240D}} > {\rm{L81A}} \sim {\rm{K154A}}{\rm{.}}$$

Although the statistics of other RMSD values give different rankings, the L126W was consistently ranked to be the most stable mutant.

### Local interactions between mutation residue and neighboring residues

According to the simulation results, the mutation affected the local interactions, as elaborated in detail as the following:

1. As shown in Fig. 5, the L81^2.46^ maintained a close distance to L127^3.42^, and a larger distance to M61^1.54^ in both wild type and L81A mutant through simulations. The L81A mutant showed smaller distance (~4 Å) between residue A81 and L127, compared to that (~5 Å) in the wild type system. The small error bars indicate stable interactions between these residues.

**Figure 5:**
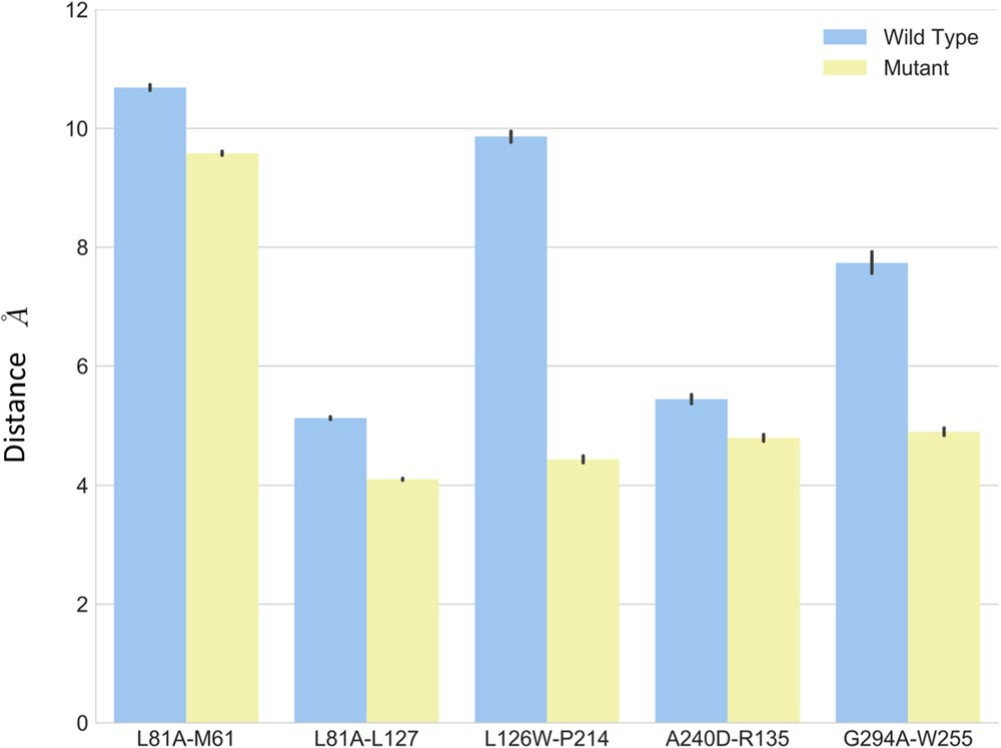
The distance from mutational residue and affecting residues for wild type (blue) and mutant (yellow).
2. The average distance between L126^3.41^ and P214^5.50^ is substantially larger than its counterpart in L126W mutant (Fig. 5). The distance is almost 9.8 Å in the wild type, and it is 4.3 Å in the mutant. The simulations results suggest that mutant L126W enhanced interactions between these two residues.
3. The residue K154^4.43^ points away from the helix bundle in the initial structures in both wild type and K154A constructs, and they remain pointing outward through the simulations, suggesting that K154A mutation did not change the local interactions.
4. As shown in Fig. 5, the distance between residue 240 and residue 135 is smaller in A240D mutant compared to that in the wild type. The A240^6.33^D and R135^3.50^ moved closer and formed salt bridge to stabilize the inter-helix packing.
5. For the G294A mutant, the distance between G294^7.32^A and W255^6.48^ was reduced from 7.5 Å in wild type to about 4.0 Å.

To summarize, the mutants resulted enhanced local interactions in most constructs, especially in the cases of L126W and G294A.

### Structure clustering analysis

The CXCR1-T4L complex may undergo substantial conformational changes during 500 ns simulations, so we carried out clustering analysis to group the structures based on their similarity. First, the pairwise RMSD was computed based on the TM domain of CXCR1; then Javis-Matrick method was used for clustering. The criteria to join any new structure to an existing cluster is as the following: there is at least one structure in the cluster share three common neighbors with the new structure, where the neighbors are 10 most similar structures or all structures within the RMSD cutoff of 1.5 Å. The clustering program implemented in Gromacs package was used for this analysis.

**Figure 6:**
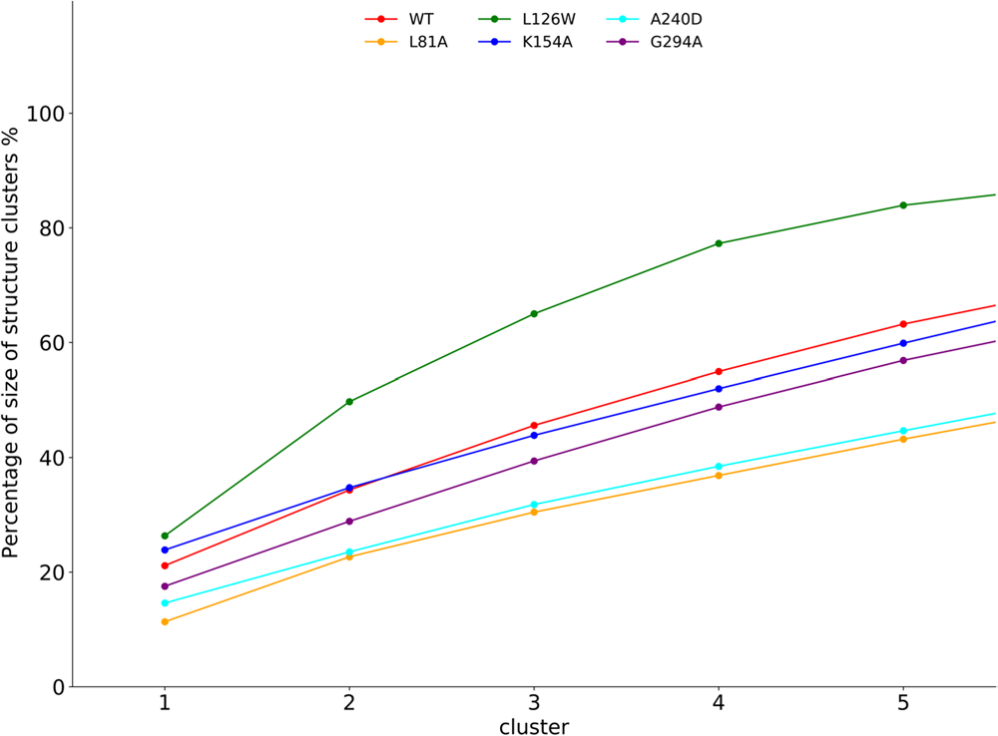
The cumulative percentage of top 5 clusters of structure ensemble obtained from simulations.

The size of each cluster is correlated to stability of the corresponding cluster. The accumulative percentages for five largest clusters are shown in Fig. 6. The structure ensemble of L126W is mostly centered around a few stable conformations as shown by the large portion occupied by the five largest clusters (up to 80%). The mutant L81A and A240D had the fewest structures represented by five most populated clusters (less than 40%). Larger cluster corresponds to deeper energy minimum, which could result in higher energy barrier for the transiting to different conformations. The L126W construct may have several deep energy minima corresponding to the largest clusters, which protect the unfolding of the protein. In contrary, the L81A and A240D may have smooth energy landscape with lower energy barriers for conformation transitions to happen easily. As it is computational challenging to carry out dynamics simulations at a broad temperature range for CXCR1-T4L complex in lipid-solvent environments, we defer the simulation of CXCR1-T4L unfolding to a future study. Based on the simulation results with the homology models, we found that the L126W mutation increased the structure populations at native or several near native conformations.

### Thermo-stability assay on CXCR mutants

**Figure 7:**
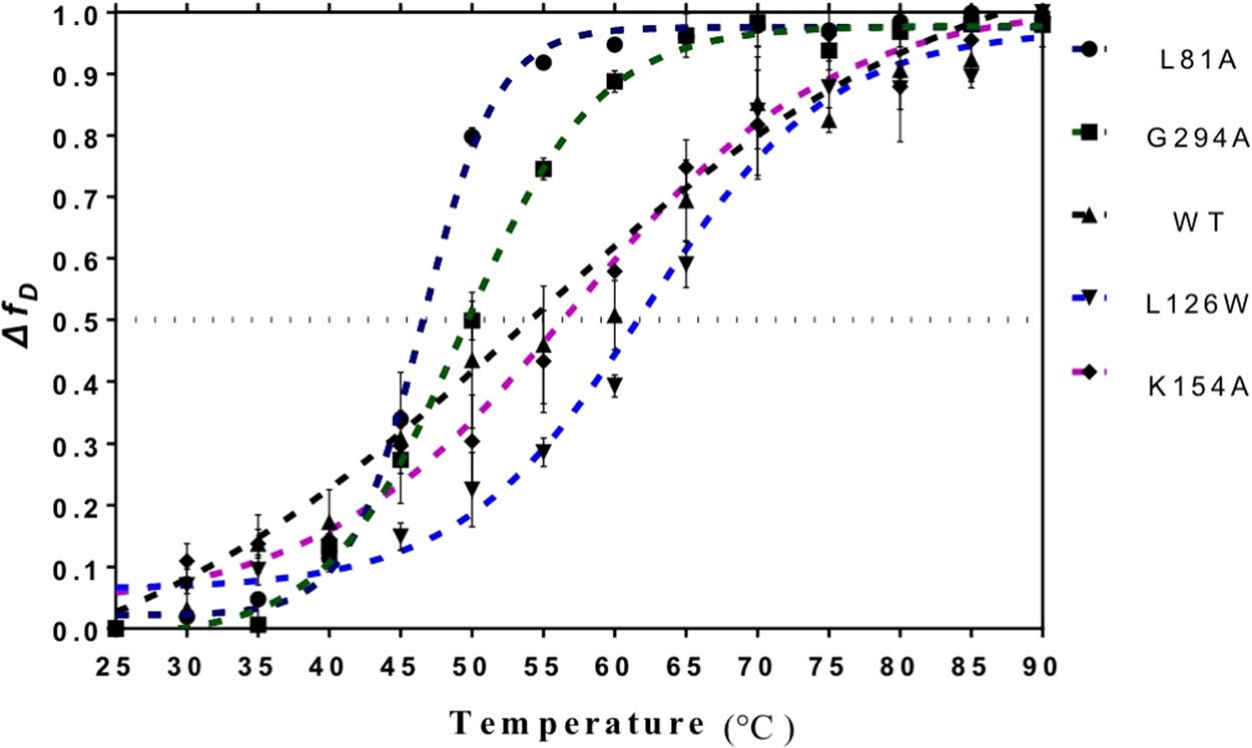
The melting curve of wild type and mutants of CXCR1 measured by the circular dichroism (CD) spectroscopy. In order to measure the T_m_, the ellipticity was measured from 25 °C to 90 °C with increasing the temperature by 5 °C, and the fraction of denatured protein (ΔfD) was calculated. The fitting results for each mutant are represented by a dash line.

Five mutants and the wild type CXCR1-T4L were expressed and purified to measure thermostability. The melting curves are shown in Fig. 7 and the estimated T_m_ values are summarized in Table 2 (see Supplementary Table S1 for the details of the data fitting). By comparing data with simulation results, we found that (1) the L126W mutant was successfully predicted to improve the thermostability of CXCR1-T4L; (2) the L81A mutant has a lower T_m_ value compared to the wild type, consistent with the simulation results. The protein expression level for mutant A240D was very low compared to the wild type, so the thermostability measurement was not conducted for the A240D construct. For the other two mutants, the values were not changed significantly (increased by 3.44 °C for K154A, and decreased by 4.42 °C for G294A).

**Table 2:** The melting temperatures of five constructs.

Construct	T_m_ (°C)	ΔT_m_ relative to wild type (°C)
Wild Type	53.78	0
L81A	46.56	−7.22
L126W	62.15	+8.37
K154A	57.22	+3.44
G294A	49.36	−4.42

The local interactions near the mutation sites, overall structure fluctuations, conformation clustering, together with the mutational assay for T_m_ value analysis suggest the following:

1. The mutant L126W is significantly more stable than wild type as predicted and experimentally validated. This construct can be a candidate for CXCR1-T4L crystallization;
2. The homology model HM-CCR2 has the prediction power on structural stability.
3. More generally, the computational modeling and dynamics simulation methods can provide useful information to guide the development of thermostable mutants to facilitate the crystallizations.

## Discussions and Conclusions

In this work, we present the results on the study of the CXCR1-T4L complex, a GPCR protein with the T4L fusion partner, using homology modeling, molecular dynamics simulation and mutagenesis experiment methods. By comparing the computational results with experimental data, we found that using CCR2-T4L crystal structure as the homology template can yield a plausible predicted structure. Secondly, both computational study and experimental measurement showed that the mutant L126W showed improved thermostability. Specifically, the mutant L126W has an increase of about 8.37 °C in melting temperature compared to the wild type, making it a potential mutant for future studies towards crystallization.

### The choice of homology models

As shown previously, the two homologous proteins CXCR4 and CCR2 both have acceptable sequence similarity compared to the target protein CXCR1 (see Fig. S1), and the two crystal structures can serve as potential templates for homology modeling. Because the CXCR4-T4L was co-crystallized with antagonist chemokine vMIP-II, making it in a different state from the apo-form; furthermore, the chemokine binding may cause the rearrangement of helices and even potentially result the TM5 helix extending to the T4L domain. Due to this reason, we selected the CCR2-T4L as the primary template in this study. On the other hand, because of the high sequence similarity between the receptors and small differences between CXCR4 and CCR2 crystal structures, we carried out a control study using CXCR4-T4L as the alternative template. The same analysis described in the previous section was carried out for the homology model based on CXCR4-T4L (briefly referred to as HM-CXCR4, details are referred to the supplementary materials). The two homology models have small structure differences in the receptor domain, so it is difficult to judge the quality of the two models from static structures. Through extensive computational simulations, it is possible to identify significant differences related to the stabilities of the wild type and five mutants. The HM-CCR2 is consistently better than the model based on CXCR4, in the analysis of both the local interactions near the mutation sites and overall thermal stability. The root of such differences is likely lying in the orientation and position of T4L relative to the CXCR1, as shown in Fig. 1. It is also noted that the HM-CXCR4 has a helical segment linking T4L and TM5 inherited from its template. The helix is more rigid than the loop linker in HM-CCR2, so the tension between T4L and CXCR1 in HM-CXCR1 structure may affect the structure and dynamics in the CXCR1 domain. It is worthwhile to mention that the hybrid approach in this study combines the structure prediction, MD simulation, and experimental studies. For validation purpose, it is easier to apply the approach to a protein with known structures, such that the uncertainty in the structure prediction is removed. However, the ultimate goal is to determine the unknown structures of proteins by designing mutants that have enhanced stability. Therefore, we applied this method to a more challenging system (the CXCR1) that requires utilizing the state-of-the-art structure predictions. The success in identifying the L126W mutant showed that the approach is very useful in protein design, although there are some disagreements in the stabilities of other mutants.

### The comparison to the NMR structure

The CXCR1 receptor structure has been determined using solid state NMR method in crystalline state. Because NMR models represent a structural ensemble, it is non-trivial to pick a single conformation as the starting model for molecular dynamics simulation, we did not carry out dynamics studies with NMR structure in this work. The first model in the NMR structure ensemble (NMR-CXCR1) was used to compare with the homology models (HM-CCR2 and HM-CXCR4). Despite the overall structure similarity to homology models, the NMR-CXCR1 structure has larger differences, as indicated by RMSD values. A detailed analysis using a new metric for GPCR protein structure comparison, 7 × 7 RMSD matrix, was also carried out to compare the three models in detail. The pairwise comparison reveals the same fact that the two homology models are similar, and they are less similar to the NMR-CXCR1 structure. Furthermore, the 7 × 7 RMSD matrix showed that the individual TM helix are very similar in three models, the arrangement of 7 helices resulted the overall structure differences. The packing of TM3, TM4, and TM5 are similar in all three models. The major difference between HM-CXCR4 and HM-CCR2 is the arrangement of TM7 relative to the TM3, TM4 and TM5. The NMR-CXCR1 structure also revealed that the binding site for the chemokine IL8 is hardly accessible, because the extracellular domain is in a closed state. It is arguable that the NMR-CXCR1 structure represents a different state of the CXCR1 receptor.

### The prospect of *in silico* construct design, simulations for experimental GPCR structure determination

Despite the development of structure determination using solid state NMR, cryogenic electron-microscopy, and X-ray crystallography including the serial crystallography with X-ray free electron lasers, the resolved structures of GPCR molecules only represent a small fraction of the GPCR family. The development of GPCRdb facilitates the usage of accumulated knowledge to guide the protein construct design. Homology modeling provides initial 3D structures that can be used to make rational choices among the numerous designs provided in the GPCRdb. For the case of CXCR1 thermostability improvement, there are 37 mutant constructs in the GPCRdb, and five were selected based on the homology modeling structure HM-CCR2. However, it is still difficult to predict the impact of mutations to the stability solely based on static structure comparisons. We extended the analysis to structure dynamics by carrying out extensive simulations using all-atom models. The stability of each mutant is inferred from the analysis of structure ensemble generated from simulations. The results from computational studies provide important information for experimental testing. Molecular dynamics simulation and analysis have been applied in quantifying the thermostability of mutants with engineered disulfide bonds^31^. Here, we demonstrated that simulation approach is applicable for point mutations in GPCR molecules. With the advancement of high performance computing, it is possible to conduct systematic MD simulations to microsecond scales with reasonable throughput. For example, using 400 CPU cores of the TianHe-2 supercomputer, it took one week to obtain microsecond simulation trajectories. The utilization of GPU acceleration will help the throughput at lower energy consumption. The analysis results from MD simulations can be used to guide the selection of more promising constructs from accumulated databases, such as the GPCRdb, for experimental studies.

In this work, we focus on the thermostability improvement by designing mutant constructs. The other important aspect of structural biology is that the introduced mutation should not affect the protein activities, so that the functions can be maintained in the mutants. The L126W will be subject to both activity measurements and crystallizations in future studies. Once the first structure of CXCR1 is obtained, it can be compared with the predicted models and for the next round of mutant construct optimization.

In summary, the structure of CXCR1-T4L complex was predicted using homology modeling methods, and several mutant constructs were designed based on database analysis. Extensive all-atom molecular dynamics simulations were used to investigate the stability variation due to the mutations, which were then validated using mutagenesis experiments. We found that the homology model based on CCR2-T4L crystal structure is highly consistent with experimental data. The L126W mutant has a significant improvement in thermostability compared to the wild type, as verified by the experiments, making it a promising target for crystallization. This work describes a pipeline for GPCR research using *in silico* design method. It is expected that the method can be generalized to other GPCR systems with experimental structures or predicted structures.

## Supplementary information

supplementary materials

GPCRdb mutation Recommendation list
