# Supplementary material for: Computer aided protein engineering to enhance the thermo-stability of CXCR1- T4 lysozyme complex
Source: Sci Rep. 2019 Mar 29;9:5317. doi: 10.1038/s41598-019-41838-2 (PMC6441008; doi:10.1038/s41598-019-41838-2)
Supplement: Supplementary file 1 — supplementary materials [file 41598_2019_41838_MOESM1_ESM.docx]

Supplementary Information for

**Computer aided protein engineering to enhance the thermo-stability of CXCR1- T4 lysozyme complex**

Yang Wang^a1^, Jae-hyun Park^b1^, Cecylia Severin Lupala^a^, Ji-Hye Yun^b^, Zeyu Jin^b^, Lanqing Huang^a^, Xuanxuan Li^a^, Leihan Tang^ac^, Weontae Lee^b^, and Haiguang Liu^a^

*^a)^ Complex Systems Division, Beijing Computational Science Research Center, Beijing 100193, China*

*^b)^ Department of Biochemistry, College of Life Science & Biotechnology, Yonsei University, Seoul, 03722, South Korea*

*^c)^ Department of Physics and Institute of Computational and Theoretical Studies, Hong Kong Baptist University, Hong Kong, China*

Correspondence email: [wlee@spin.yonsei.ac.kr](mailto:wlee@spin.yonsei.ac.kr), [hgliu@csrc.ac.cn](mailto:hgliu@csrc.ac.cn)

^1^These authors contributed equally

This document includes four sections: (1) the sequence comparison between CXCR1, CXCR4 and CCR2; (2) the three experimentally determined structure comparison; (3) the analysis on the stability of CXCR1-T4L using simulations starting with the homology structure predicted based on CXCR4-T4L; and (4) the experimental results for protein expression levels and thermostability analysis.

1. **Sequence Similarity between CXCR1 and two templates CXCR4 and CCR2**

The receptors in the Chemokine receptor family have high sequence similarity, especially in trans-membrane (TM) regions. The sequence similarity between CXCR1 and CXCR4 is about 38% for full sequence and about 42% for transmembrane regions; while CXCR1 and CCR2 share a sequence similarity of about 33% for full sequence (about 38% for transmembrane regions). Both CXCR4 and CCR2 were crystalized with fusion partner T4 lysozyme T4L. The sequence alignments for transmembrane domains are shown in Figure S1.

1. **Structure comparison between NMR structure of CXCR1 and homology templates**

The 7x7 RMSD matrices were calculated to quantity the structure differences between these models. As shown in Figure S2, the NMR-CXCR1 structure exhibits larger differences compared to the other two structures solved using crystal diffraction method. The individual helix has small structural differences, but the packing in NMR-CXCR1 structure shows pronounced differences compared to the crystal structures of CXCR4-T4L and CCR2-T4L.

1. **The results from homology models based on CXCR4-T4L structure**

As a control, using the same methods described in the main text, we studied the stability and mutation effects with a different homology model HM-CXCR4 that is based on the CXCR4-T4L without the viral chemokine antagonist ligand vMIP-II.

As shown in section 3 in the main text, the stability of each construct (wild type and mutants) of HM-CXCR4 was investigated using all-atom molecular dynamics simulations. For each system, three independent 500 ns simulation trajectories were generated, resulting a structure ensemble of 1,500 structures for detailed analysis. The analysis includes computing RMSD with respect to the average structure in each ensemble to quantified structural fluctuation, inspecting the interactions between the key amino acids at mutation sites and their neighboring residues and clustering analysis of the structural ensemble.

**3.1 The conformational fluctuations**

The RMSD analysis results were summarized in Fig.S3 for all six constructs. The definition of RMSD^TM^, RMSD^T4L^, RMSD^ALL^ and RMSD^T4L’^ results suggested that the CXCR1 trans-membrane domain were stable for all constructs, with most median values of RMSD between 2Å and 3Å. Among the five mutant constructs, the A240D construct showed the smallest fluctuations in the simulations starting with HM-CXCR4 structures (Fig. S3). Unlike the HM-CCR2 model, the motions of T4L relative to CXCR1 of HM-CXCR4 were observed to be smaller than that in the wild type. This might be due to the extended helix between T4L and H5 of CXCR1 receptor. Based on the RMSD^TM^ statistics, the ranking of stability can be summarized as the following:

A240D>G294A~L81A>WT~K154A>L126W.

**3.2 Local interactions between mutation residue and neighboring residues**

According to the simulation results of HM-CXCR4 construct, the local interaction affected by point mutations were elaborated in detail as the following:

(1) The L81^2.46^ maintained a close distance to L127^3.42^, and a larger distance to M61^1.54^ in both wild type and L81A mutant for HM-CXCR4. The mutant showed smaller distance between residue A81 and L127.

(2) The average distance between L126^3.41^ and P214^5.50^ is smaller in L126W mutant than that in the wild type. In HM-CXCR4, the distance is about 6.2 Å in the wild type, while the distance is about 3.7 Å in L126W mutant.

(3) The residue K154^4.43^ points towards away from the helix bundle in the initial structure. Through the simulations, both wild type and K154A have their side chains remained pointing outward. Therefore, the mutation from lysine to alanine did not change the local interactions for this residue.

(4) The distance between residue 240 and residue 135 is smaller in A240D mutant compared to that in the wild type. In the simulations starting with HM-CCR2 construct, the A240^6.33^D and R135^3.50^ moved closer and formed salt bridge to stabilize the inter-helix packing. However, this was not observed for the simulation with HM-CXCR4 model, in which the distance was larger than 7.0 Å.

(5) For the G294A mutant, the two homology models also showed different trends, the distance between G294^7.32^A and W255^6.48^ was reduced from 7.5 Å in wild type to about 4.0 Å using HM-CCR2 as starting model (Figure 5 in main text). The HM-CXCR4 model showed opposite trend, increased from 4.0 Å to 6.5 Å (Fig. S4).

**3.3 Structure clustering analysis**

The accumulative percentages for five largest clusters were shown in Fig. S5. For the structure ensembles generated from HM-CXCR4 model, the largest clusters for six constructs account for about 20% to 30%, and the five largest clusters account for 55% to 80% of all structures in the ensembles generated from simulations. As shown in Fig. S6, the larger accumulative percentages (~80% in the five largest clusters) of mutants L81A and L126W suggest that these two mutants exhibit smaller structure variations. The mutant K154A has more diverse conformations, as the five largest clusters only include about 55% of structures.

1. **The expression level for mutant A240D compared to those of wild type and K154A**

The expression level for A240D is very low (about 5% of the wild type expression level under the same conditions, Figure S6), so it is difficult to prepare large quantity of sample for thermostability measurement, nor for future crystallization.

**
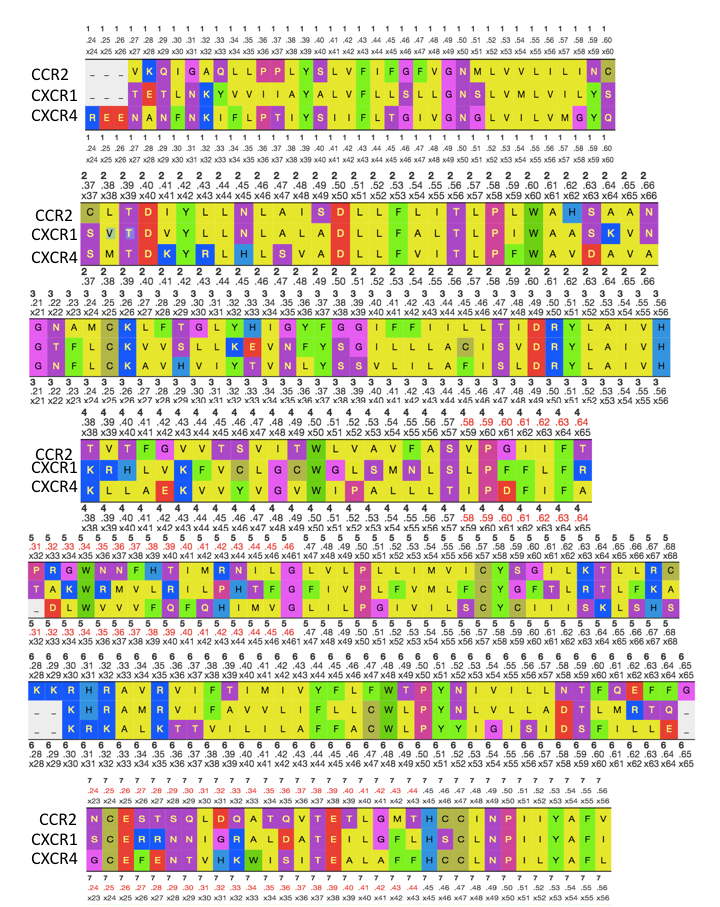
**

**Fig. S1** Sequence alignment of CCR2, CXCR1 and CXCR4 in TM1-TM7 regions.

**
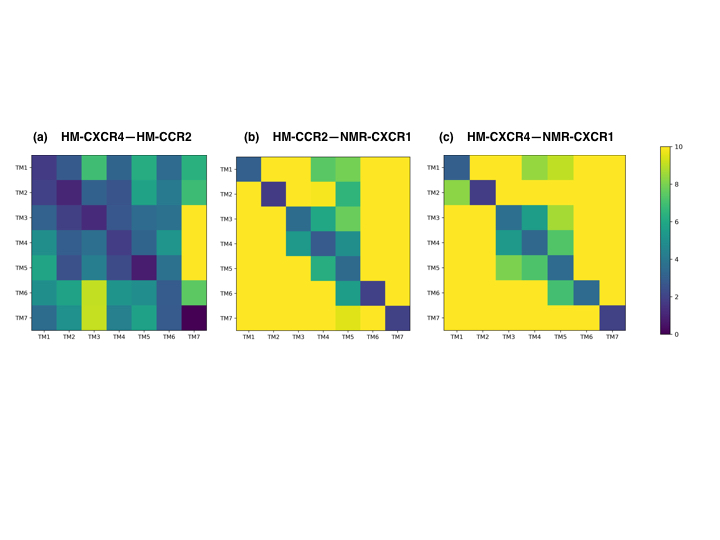
**

**Fig. S2 7x7 RMSD matrices between three structures: NMR-CXCR1, HM-CXCR4, HM-CCR2.** (a) 7x7 RMSD matrix between HM-CXCR4 and HM-CCR2. (b) 7x7 RMSD matrix between HM-CCR2 and NMR-CXCR1. (c) 7x7 RMSD matrix between HM-CXCR4 and NMR-CXCR1. The numbers next to the colorbar indicate the RMSD values (in the unit of Å).


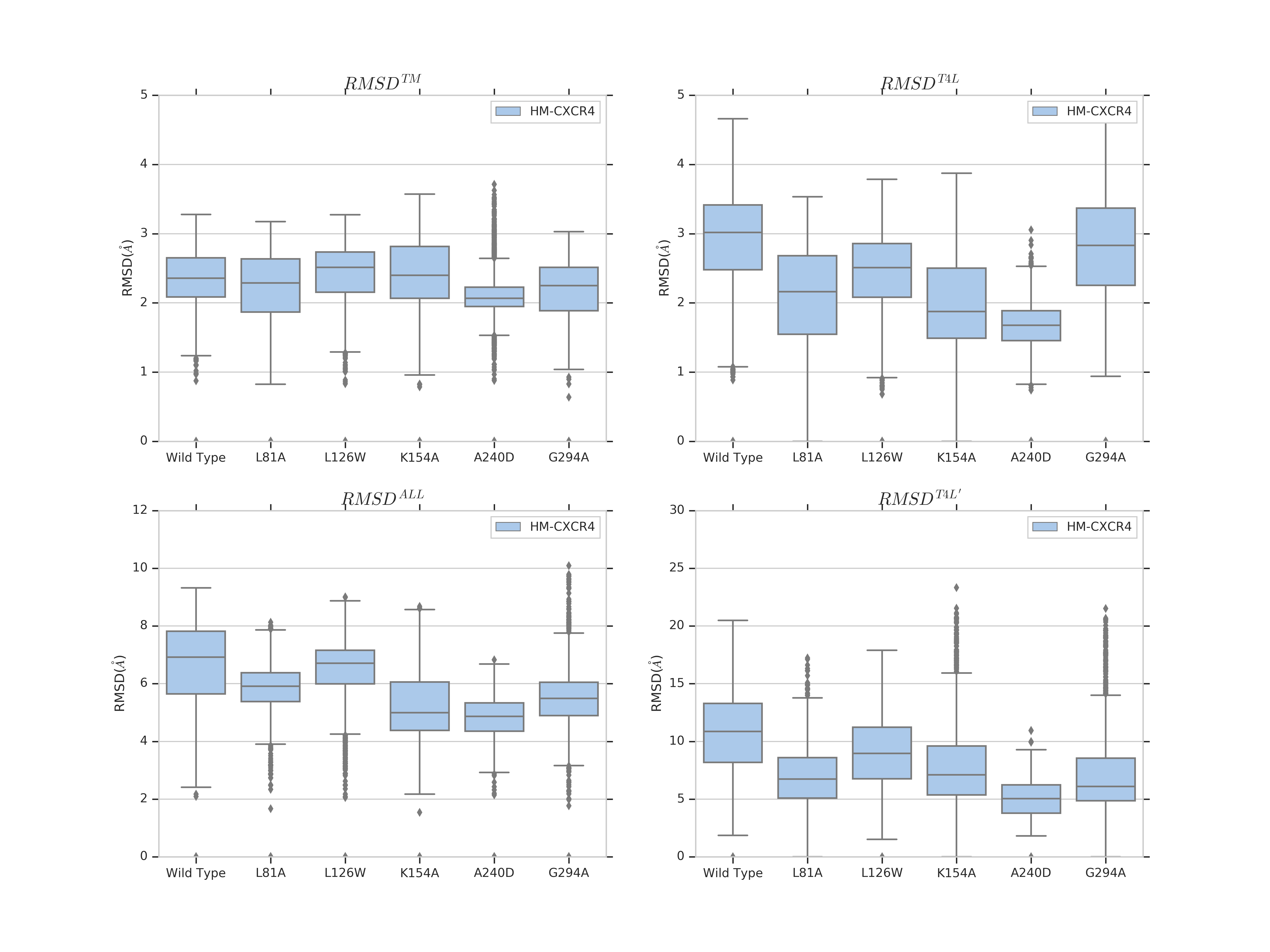


**Fig. S3** The RMSD^TM^, RMSD^T4L^, RMSD^ALL^ and RMSD^T4L’^ from simulations using HM-CXCR4 structures as initial conformations (see main text for details).


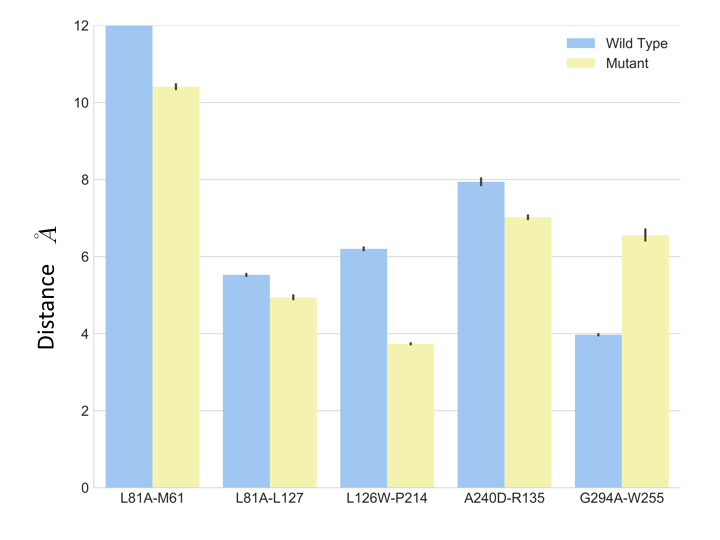


**Fig. S4** The distance from mutational residue and affecting residues for wild type (blue) and mutant (yellow), based on simulation results using HM-CXCR4.





**Fig. S5** The cumulative percentage of top 5 clusters of structure ensemble obtained from simulations with HM-CXCR4.

a.


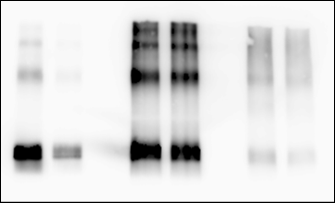


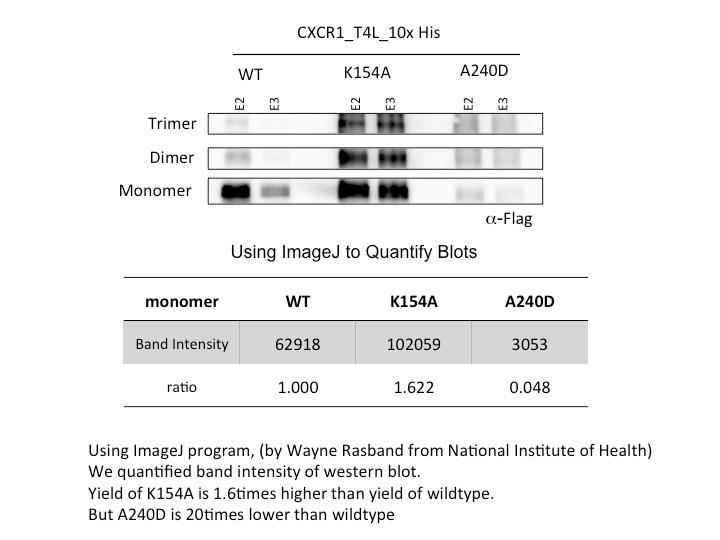


b.

**Fig. S6 The expression levels of wild type, K154A, and A240D constructs.** (a) The original gel with full length. (b) the protein segments were selected for clear presentations. The K154A has about 1.6 times higher yield compared to the wild type, and the A240D is about 20 times lower than the wild type. The expression levels for other mutant constructs are comparable to that of the wild type.

**Table S1. The fitting to the circular dichroism data.**

| Construct | L81A | G294A | WT | L126W | K154A |
| --- | --- | --- | --- | --- | --- |
| R square | 0.998 | 0.995 | 0.9656 | 0.9791 | 0.973 |
| Hill-Slope | 0.1674 | 0.09136 | 0.0279 | 0.06631 | 0.04762 |
| Tm (℃) | 46.56 | 49.36 | 53.78 | 62.15 | 57.22 |
| ΔTm relative to wild type (℃) | -7.22 | -4.42 | 0 | 8.37 | 3.44 |
