# Supplementary material for: Computer aided protein engineering to enhance the thermo-stability of CXCR1- T4 lysozyme complex
Source: Sci Rep. 2019 Mar 29;9:5317. doi: 10.1038/s41598-019-41838-2 (PMC6441008; doi:10.1038/s41598-019-41838-2)
Supplement: Supplementary file 2 — GPCRdb mutation Recommendation list [file 41598_2019_41838_MOESM2_ESM.pdf]

Column1	Generic position	Mutation	hits	methods	Column2	Column3	Column4
1	7x45	H297N, H297N	2	struc_rules_inactive:Sodium ion site Addition,cons_strucs			
2	3x39	G124S, G124S	2	struc_rules_inactive:Sodium ion site Addition,cons_strucs			
3	5x61	T225I, T225I	2	cons_strucs,cons_rf			
4	7x46	S298C, S298C	2	cons_rf_and_class,cons_rf			
5	3x41	L126W, L126W	2	termo_Common mutation (Mut),termo_Common mutation (Wt)			
6	7x41	G294A, G294A	2	cons_rm_nonconserved_GP,cons_rf			
7	4x42	V153A	1	cons_strucs			
8	34x50	A142P	1	cons_strucs			
9	2x52	L87L	1	termo_Common mutation (Mut)			
10	6x37	I244A	1	termo_Common mutation (Mut)			
11	3x49	D134A	1	termo_Common mutation (Mut)			
12	5x58	Y222A, Y222F	1	termo_Common mutation (Wt)			
13	1x30	L37F	1	cons_rf			
14	1x36	I43P	1	cons_rf			
15	8x54	F316L	1	cons_rf			
16	1x34	V41F	1	cons_rf			
17	1x35	V42L	1	cons_rf			
18	7x52	I304L	1	cons_rf			
19	1x46	S53G	1	cons_rf			
20	2x59	I94F	1	cons_rf			
21	7x36	A289V	1	cons_rf			
22	4x53	S164A	1	cons_rf			
23	7x30	I283L	1	cons_rf			
24	3x32	K117Y	1	cons_rf			
25	5x49	V213L	1	cons_rf			
26	7x53	Y305L	1	termo_Single common mutation (Wt)			
27	3x40	I125V	1	termo_Single common mutation (Wt)			
28	2x46	L81A	1	termo_Single common mutation (Wt)			
29	4x43	K154A	1	termo_Single common mutation (Wt)			
30	6x33	A240D	1	termo_Single common mutation (Wt)			
31	6x41	V248A	1	termo_Single common mutation (Wt)			
32	5x461	G210A	1	cons_rm_conserved_G			
33	3x21	G106A	1	cons_rm_conserved_G			

34	1x49	G56A	1	cons_rm_conserved_G
35	8x47	G309A	1	cons_rm_conserved_G
36	6x49	L256N	1	struc_rules_inactive:State switch N6x49 Addition
37	6x30	K237E	1	struc_rules_inactive:Ionic lock (D/ERY) Addition
